# Physical complaints and emotional symptoms in the context of series watching: a multicenter latent profile and network analysis in Brazil and Canada

**DOI:** 10.3389/fpsyg.2026.1873349

**Published:** 2026-07-24

**Authors:** Gilvano Amorim Oliveira, Cláudio Romualdo, Wanderlei Abadio de Oliveira, Laura Soares da Silva, Luciana Bertoldi Nucci, Adriana Scatena, Denise De Micheli, Hyoun S. Kim, Makilim Nunes Baptista, André Luiz Monezi Andrade

**Affiliations:** 1 School of Life Sciences, Pontifical Catholic University of Campinas, Campinas, Brazil; 2 Psychology Section, Santo André Medical Center, Santo André, Brazil; 3 Federal University of São Paulo, São Paulo, Brazil; 4 Department of Psychology, Toronto Metropolitan University, Toronto, ON, Canada

**Keywords:** emotional symptoms, latent profile analysis, physical complaints, series watching, smartphone use

## Abstract

**Introduction:**

Few studies have examined the physical complaints, emotional aspects, behavioral indicators, and quality of life associated with series watching (PCSW) across cultural contexts.

**Methods:**

This multicenter, cross-sectional study investigated latent profiles of PCSW and their associations with emotional variables, smartphone addiction, food addiction, quality of life, and series-watching intensity (SWI) among university students in Brazil (*n* = 1,190) and Canada (*n* = 306). Participants completed five author-developed PCSW self-report items, used as an operational measure rather than a validated scale, together with standardized measures of smartphone addiction (SAS-SV), emotion dysregulation (DERS-18), emotional symptoms (DASS-21), impulsivity (UPPS-P), food addiction (mYFAS 2.0; AEBS), and quality of life (WHOQOL-Bref).

**Results:**

A latent profile analysis identified three profiles characterized by high (HPC), moderate (MPC), and low (LPC) levels of physical complaints. The HPC profile exhibited poorer emotional and behavioral indicators, higher levels of smartphone and food addiction, lower quality-of-life scores, and greater SWI. Spearman correlations revealed positive relationships between PCSW, emotional symptoms, emotion dysregulation, problematic smartphone use, and food addiction indicators, as well as negative relationships with quality of life. Network analysis indicated that depression, stress, and emotion-regulation difficulties formed a central core, with PCSW showing more peripheral connections to this core, particularly in relation to problematic smartphone use and emotion regulation. These connections were broadly similar across countries, with slightly stronger associations in Brazil and greater importance of addictive eating indicators in Canada.

**Discussion:**

Our findings indicate that emotional and technological factors are associated with PCSW, underscoring the importance of emotion regulation, smartphone use, and SWI for students' wellbeing.

## 1 Introduction

The growing popularity of streaming platforms, driven by increased internet access, has significantly facilitated the consumption of series across age and socioeconomic groups, enabling quick, flexible, on-demand access *via* devices such as smartphones, tablets, and computers (Kaur and Ashfaq, 2023). This shift has led to more frequent and prolonged consumption of films and series, increasing screen time at home, at work, during commutes, and in leisure settings (Wu et al., 2025). In countries such as Brazil and Canada, this digital landscape has achieved high penetration. In Brazil, 93.6% of households had internet access in 2024, and 32.7 million households had paid subscriptions to video streaming services (Brazilian Institute of Geography Statistics (IBGE), 2025). In Canada, official industry reports indicate a more consolidated ecosystem: the proportion of households exclusively connected to streaming increased from 23% in 2023 to 29% in 2024 (Canadian Radio-television and Telecommunications Commission (CRTC), 2025).

This contrast between the two countries suggests that widespread connectivity does not uniformly explain how individuals from diverse sociodemographic and cultural contexts engage in series watching. More intense and prolonged series-watching patterns have also been associated with the reward system, reduced inhibitory control, executive functioning, and behavioral regulation—features common in behavioral addictions and substance use (Brand et al., 2025; Paschke et al., 2022). Accordingly, this series-watching pattern has attracted increasing research attention, particularly since 2013, as streaming platforms consolidated and audiovisual consumption patterns shifted (Flayelle et al., 2020). In this context, terms such as “marathon viewing” and, particularly, “binge-watching” began to be used widely to describe watching multiple episodes of the same series, either sequentially or in a single session (Cha and Chan-Olmsted, 2024; Viens and Farrar, 2021). Despite its widespread use, no consensus exists regarding the definition of “binge-watching.” For example, a systematic review reported variability across studies in the number of episodes considered, session duration, behavior frequency, and content type (Flayelle et al., 2020). Furthermore, binge-watching is not part of any formal diagnostic category in the main mental health classification systems and is better conceptualized as an evolving theoretical and behavioral construct (Alimoradi et al., 2022; Bastos et al., 2024; Paschke et al., 2022). Therefore, in the present study, series-watching intensity (SWI) refers to the frequency and duration of consecutive episode viewing, whereas intensive series watching (ISW) is an operational behavioral classification rather than a clinical diagnosis.

In this context, series-watching intensity (SWI) can be examined without relying on a rigid diagnostic definition; therefore, it may provide relevant evidence on its health-related correlates (Ameri et al., 2024; Chen et al., 2024; Pittman and Steiner, 2021). For example, growing evidence links series-watching to physical complaints (PCSW), particularly eye pain, blurred vision, tearing, dry eyes, and headaches (Allwihan et al., 2024; Kaur et al., 2022; Pucker et al., 2024). In the psychological domain, studies have associated SWI with symptoms of anxiety, depression, stress, and loneliness (da Silva et al., 2025; Hamza et al., 2023; Raza et al., 2021). SWI has also been linked to sleep problems and sedentary lifestyles (Srinivasan et al., 2021). Furthermore, studies link SWI to emotion-regulation difficulties and impulsivity, especially when consumption is more intense and frequent (Starosta et al., 2021).

Impulsivity, in turn, is linked to difficulties with emotion regulation. Individuals with fewer resources for managing negative emotions are more likely to engage in behaviors that provide immediate gratification to relieve discomfort. This tendency is widely associated with excessive digital media use (Castro et al., 2021; Diotaiuti et al., 2022; Steins-Loeber et al., 2020). Other important links with SWI include compulsive eating patterns. A meta-analysis of 23 studies found that consuming audiovisual content while eating increases calorie intake and reduces awareness of how much is consumed (Garg et al., 2025). Other researchers have reported similar results, showing that TV viewing is associated with excessive intake of ultra-processed, hyperpalatable foods high in sugar and fat (Aghababian et al., 2021; De Oliveira Martins et al., 2024; Dejavitte et al., 2025). In this study, smartphone addiction and food addiction are treated as dimensional indicators of addictive-like behaviors, not as formal clinical diagnoses.

Regarding health, prolonged screen exposure facilitated by SWI has significant clinical implications, including circadian rhythm disruption and text neck syndrome, characterized by sustained neck flexion and a downcast gaze while using mobile devices (Lin and Su, 2025). Smartphones are among the most common devices for streaming series because of their portability and ease of connection, enabling users to install apps from streaming platforms (Starosta et al., 2020). This accessibility may facilitate more frequent series watching and may co-occur with extended smartphone use, a pattern associated with health-related risks (Sever and Özdemir, 2021; Younis, 2023). Nevertheless, gaps remain in the literature regarding the relationship between SWI and excessive smartphone use.

Despite these emotional and behavioral considerations, few studies have examined PCSW in an integrated manner. A recent systematic review of the association between dry eye symptoms/digital eyestrain and mental health indicators included 15 studies, of which eight assessed depression, seven assessed anxiety, and seven assessed stress. The review found predominantly positive associations between higher frequency and intensity of ocular symptoms and poorer emotional outcomes (Kopilaš et al., 2025). In some studies, these associations ranged from modest to moderate positive correlations (e.g., *r* > 0.04; *p* < 0.001), whereas others observed stronger relationships between the intensity of ocular symptoms and depression (*p* < 0.001; *r* > 0.5), as well as consistent associations with anxiety and stress (*p* < 0.001; *r* = 0.4). Furthermore, among students, approximately one-third reported at least six ocular symptoms, and approximately 30% of medical students reported more severe symptoms.

These findings indicate that symptoms such as eye pain, dryness, blurred vision, and headache may be part of a broader profile of psychological and behavioral vulnerability associated with intense digital consumption patterns (Chen et al., 2024). This issue is particularly relevant when investigating individuals from countries with distinct sociodemographic profiles and cultural contexts, such as Brazil and Canada. Comparing these two groups can help assess whether the associations between PCSW and the study's emotional and behavioral variables hold across different contexts. This is important because it helps assess whether the relationship between PCSW and variables such as emotional distress, emotional dysregulation, impulsivity, and problematic smartphone use persists even among groups embedded in distinct sociocultural realities. Due to the study's cross-sectional and multicenter design, these comparisons should be viewed as exploratory and descriptive rather than as proof of cross-cultural measurement equivalence.

Advancing this scientific field requires statistical models that effectively capture the heterogeneity of these indicators. This study hypothesizes that symptoms such as eye pain, blurred vision, tearing, dry eyes, and headache are heterogeneously distributed and may constitute distinct physical complaints with varying levels of emotional distress and behavioral vulnerability (Basilious et al., 2021). Because PCSW was assessed using author-developed items, these indicators are treated as an operational measure of self-reported physical complaints during series watching. Accordingly, latent profile analysis (LPA) is effective at identifying subgroups with similar response patterns (Ren et al., 2021; Tóth-Király et al., 2017). Recent person-centered research has employed LPA to identify digital behavior profiles linked to distress, fear of missing out, and self-esteem, highlighting the importance of profile-based methods in this area (Aslan and Koç, 2026). Complementarily, statistical models such as network analysis also allow us to examine how these physical complaints relate to variables such as anxiety, depression, stress, emotion regulation, impulsivity, problematic smartphone use, dysfunctional eating patterns, and quality of life, thereby enhancing understanding of how these phenomena are organized in the series-watching context (Flayelle et al., 2019).

This study aimed to identify latent profiles of PCSW and to examine how these profiles differ across emotional variables, problematic smartphone use, dysfunctional eating patterns, quality of life, and SWI indicators among university students from Brazil and Canada. The study focused on three key questions: first, how many distinct profiles would emerge from the PCSW items; second, whether these profiles would differ in emotional symptoms, problematic smartphone use, indicators of addictive-like eating, quality of life, and SWI; and third, whether the patterns of these associations would be generally similar across the Brazilian and Canadian samples. We hypothesized that (1) LPA would identify three or more PCSW profiles, differentiated by the intensity and pattern of physical complaints associated with watching series. (2) Profiles with higher PCSW levels would show greater emotion regulation difficulties; more anxiety, depression, and stress symptoms; greater problematic smartphone use; more dysfunctional eating patterns; and poorer quality of life. (3) Profiles with higher PCSW levels would also be associated with greater intensity of watching series.

## 2 Methods

### 2.1 Study design and sample characteristics

This study employs a quantitative, cross-sectional design using convenience and non-probabilistic sampling methods. The study participants (*N* = 1,496) included 1,190 Brazilian and 306 Canadian university students. All participants completed an online questionnaire. Data were collected from a multicenter study conducted across three Brazilian universities and one Canadian university. Participants were recruited in university environments through direct contact with students, classroom announcements, and online survey access. The survey link was provided electronically, and a QR code was also distributed to make accessing the online form easier.

Eligibility criteria required participants to be actively enrolled university students in Brazil or Canada, aged 18 or older, and to agree to participate by giving informed consent before completing the questionnaire. Exclusion criteria included incomplete responses, duplicate entries, or refusal to participate. During the initial screening, 195 individuals were excluded: 144 from Brazil and 51 from Canada, due to incomplete or duplicate responses, or non-participation. The final sample for this study consisted of 1,496 participants-−1,190 from Brazil and 306 from Canada.

The Brazilian participants filled out the questionnaire in Portuguese, while the Canadian participants completed it in English or French. Due to the significant difference in sample sizes between Brazil and Canada, all analyses specific to each country were interpreted with caution, particularly the network models.

The study received ethical approval in Brazil from the Research Ethics Committee of the Pontifical Catholic University of Campinas (CAAE: 55661222.9.0000.5481; approval number: 5.611.237), and in Canada from the Ryerson University Research Ethics Board (REB 2021-266). All participants gave informed consent before taking the questionnaire.

### 2.2 Measures

#### 2.2.1 Sociodemographic questionnaire

Includes factors such as marital status, gender, income, undergraduate course type, university type, and age. The country of data collection was also recorded and used in descriptive and country-specific analyses.

#### 2.2.2 Measures of series watching

Two questions assessed the series-watching behavior (Cha and Chan-Olmsted, 2024; Viens and Farrar, 2021). These include (i) SW1: Frequency of watching three or more consecutive episodes in the past 3 months, with responses ranging from “never” to “every day” or “almost every day”; and (ii) SW2: Average number of consecutive episodes watched per session, asked as “When you watch a series, on average, how many consecutive episodes do you usually watch?” In this question, participants could report values between 0 and 10, or more than 10 episodes.

#### 2.2.3 Series-watching pattern

Based on combined responses to these two questions, we defined a series-watching pattern by intensity level. Participants who reported watching three or more consecutive episodes weekly or daily/almost daily over the past 3 months, and who reported watching, on average, three or more episodes per session, were classified as Intensive Series Watchers (ISW). All other participants were classified as Less Intensive Series Watchers (LSW). This group definition was adopted from prior literature (Ameri et al., 2024; Flayelle et al., 2020), given the absence of specific scales to measure the intensity of series watching in Brazil and Canada. Furthermore, these criteria were selected to provide a more conservative description of this behavioral pattern, without implying a formal diagnostic criterion, aligning with the study's purpose.

#### 2.2.4 Physical complaints associated with series watching (PCSW)

As no tool for measuring PCSW in Brazil and Canada exists, we designed a set of questions based on constructs closely related to those in the literature (Allwihan et al., 2024; Kaur et al., 2022; Pucker et al., 2024). Participants answered five questions related to Physical Complaints (PC) on a Likert scale from “no/none” (1) to “yes/quite a bit” (5). Physical Complaints 1 (PC1: When watching a series, do you feel pain in your eyes?). Physical Complaints 2; (PC2: When watching a series, do you usually feel that your vision becomes blurry?); Physical Complaints 3 (PC3: When watching a series, do you usually feel that your eyes water?). Physical Complaints 4 (PC4: When watching a series, do you usually feel that your eyes become drier?). Physical Complaints 5 (PC5: When watching a series, do you usually get headaches?).

The PCSW score was obtained by summing the five items, with higher scores indicating more frequent or severe self-reported physical complaints related to series watching. Since these items were created by the authors and are not part of an established, validated scale, PCSW is considered an operational measure rather than a formal diagnostic or clinical tool. Internal consistency was calculated only for the total sample, with coefficients of α = 0.65 and ω = 0.62; country-specific reliability coefficients were not estimated.

#### 2.2.5 WHOQOL-bref

This tool measures quality of life using 26 items across four domains: physical, psychological, social relationships, and environment. A higher score indicates a better quality of life. This scale (Fleck et al., 2000) showed high overall internal consistency (α = 0.90).

#### 2.2.6 Modified yale food addiction scale 2.0 (mYFAS 2.0)

This tool aims to evaluate behaviors commonly linked to food addiction using a 13-item Likert scale. The items were developed based on the substance-use disorder criteria from the Diagnostic and Statistical Manual of Mental Disorders (DSM-5), adapted to eating behaviors, and the scale has a single-factor structure. Based on the scores, participants' food addiction behavior can be classified as mild (2–3 diagnostic criteria or symptoms), moderate (4–5 criteria or symptoms), or severe (six or more criteria or symptoms). The scale, adapted and validated in Brazil (Nunes-Neto et al., 2018), demonstrated high internal consistency (α = 0.89). The Addiction-like Eating Behavior Scale (AEBS) was designed to measure addiction-like eating patterns and comprises 15 Likert-scale items that assess two main aspects: appetitive drive (the urge to eat) and control over eating (the ability to regulate food intake). Higher scores indicate more severe symptoms. The AEBS (Cardoso et al., 2020) showed high internal consistency (α = 0.91). In the present study, mYFAS 2.0 and AEBS scores were interpreted as indicators of addictive-like eating patterns rather than as clinical diagnoses.

#### 2.2.7 Smartphone addiction scale—short version (SAS-SV)

This scale measures smartphone addiction by evaluating factors such as daily life disruption, positive anticipation of use, withdrawal, tolerance, dependence, and overuse. The SAS-SV includes 10 items on a Likert scale, with a total score ranging from 10 to 60; higher scores indicate more severe symptoms. The SAS-SV was adapted and validated (Andrade et al., 2020), showing good reliability (α = 0.81). SAS-SV scores were interpreted as dimensional indicators of problematic smartphone use rather than as evidence for a formal clinical diagnosis.

#### 2.2.8 Difficulties in emotion regulation scale short version (DERS-18)

This tool evaluates emotion regulation difficulties using 18 items, rated on a 5-point Likert scale from 1 (almost never applies to me) to 5 (almost always applies to me). The DERS-18 includes six dimensions: (1) non-acceptance of negative emotional responses; (2) difficulty engaging in goal-directed behaviors when experiencing negative emotions; (3) difficulty controlling impulsive behavior amid negative emotions; (4) limited access to perceived effective emotion regulation strategies; (5) lack of emotional awareness; and (6) lack of emotional clarity. Higher scores indicate greater difficulties with emotion regulation. The Brazilian version was adapted and validated (Machado et al., 2020), with reliability coefficients ranging from α = 0.86 to α = 0.93.

#### 2.2.9 Depression, anxiety, and stress scale (DASS-21)

This scale comprises 21 items divided into three subscales—depression, anxiety, and stress—each with seven items. The DASS-21 uses a 4-point Likert scale (0–3) for the last 7 days; the higher the score, the more severe the symptoms. The scale showed good internal consistency (Vignola and Tucci, 2014) for all analyzed domains (α = 0.92 for depression, α = 0.86 for anxiety, and α = 0.90 for stress).

#### 2.2.10 Short impulsive behavior scale (SUPPS-P)

This tool measures impulsivity across five areas: negative urgency, positive urgency, sensation seeking, lack of perseverance, and lack of premeditation. In this shorter version, the UPPS-P has 20 items on a 4-point Likert scale, with higher scores indicating lower impulsivity (inverted scores). The scale demonstrated good internal consistency, with alpha values ranging from α = 0.79 to α = 0.89 (Pompeia et al., 2018). Thus, lower SUPPS-P scores indicate higher impulsivity-related indicators.

### 2.3 Data analysis

All analyses were conducted using JASP version 0.95.4. The significance threshold was set at *p* < 0.05. Before the main analyses, descriptive statistics were reviewed, and the distributions of the study variables were examined. Since multiple outcomes were evaluated, *p*-values were assessed based on the consistency of observed patterns across different variables. No formal correction for multiple comparisons was used; thus, the inferential findings should be viewed as exploratory and interpreted with caution.

The LPA was conducted using questions related to Physical Complaints (PC). Specifically, the five PCSW items served as profile indicators. To determine the optimal number of profiles, multiple fit indices were used: Log-likelihood (which measures the probability of the data under the model), Akaike Information Criterion (AIC), Corrected Akaike Information Criterion (CAIC), and Bayesian Information Criterion (BIC). Additionally, entropy was used as an indicator of classification accuracy, with values closer to one indicating greater clarity and separation between latent groups (Paschke et al., 2022; Ren et al., 2021). The final solution was selected by jointly considering statistical fit, entropy, parsimony, theoretical interpretability, and the substantive differentiation of the profiles.

Based on the identified three-profile latent model, we assessed the normality of the data and the homogeneity of variances using the Kolmogorov-Smirnov and Levene's tests, respectively. Given these assumptions, we conducted a one-way analysis of variance (ANOVA) with Welch's correction to examine significant differences in sociodemographic, emotional, and digital media use variables across the groups. To identify specific differences between pairs of groups, we used the Games-Howell *post hoc* test. When available, effect sizes were considered to support the interpretation of statistically significant differences.

We conducted an exploratory correlation analysis using Spearman's correlation coefficient to identify the primary relationships among the study variables. We presented the correlation matrices as heat maps for easier interpretation. Next, we conducted an exploratory Network Analysis (NA) using the Graphical Least Absolute Shrinkage and Selection Operator (GLASSO) method. We employed the EBICglasso estimator to build a weighted, signed network, setting the adjustment parameter to 0.5 and excluding missing pairwise data. To evaluate the importance of each variable, we drew on existing studies (Pereira et al., 2024; Vitta et al., 2025) and considered four normalized centrality indices: strength, closeness, betweenness, and expected influence. Because no formal bootstrap stability analysis was performed, we interpreted the network findings with caution and described them carefully, especially for country-specific networks and for centrality indices that may be more affected by sample size.

## 3 Results

An analysis of the LPA adjustment criteria supported retaining a three-profile solution. The data showed a gradual increase in LogLik as the profile count increased (Table 1). Although AIC, CAIC, and BIC decreased progressively with additional profiles, the three-profile solution was selected because of its parsimony and theoretical interpretability, along with adequate entropy and clearly distinguishable profiles corresponding to high, moderate, and low levels of PCSW. Therefore, the three latent profiles were named High Physical Complaints (HPC), Moderate Physical Complaints (MPC), and Low Physical Complaints (LPC).

**Table 1:** Criteria for the best fit index of the latent profile analysis (LPA) model.

Profiles	Log-likelihood	AIC	CAIC	BIC	Entropy
1	−14333	28690	28766	28754	1.000
2	−13172	26383	26503	26484	0.872
3	−12867	25785	25949	25923	0.888
4	−12602	25270	25479	25446	0.896
5	−12558	25197	25449	25409	0.901
6	−12139	24371	24668	24621	0.902

LPA, latent profile analysis; AIC, akaike information criterion; CAIC, corrected akaike information criterion; BIC, Bayesian information criterion. Lower AIC, CAIC, and BIC values indicate better model fit, whereas higher entropy values indicate better classification quality.

Table 2 shows notable differences among the three profiles across all five PCSW indicators (PC1–PC5; *p* < 0.001). A clear pattern emerged: the HPC profile had the highest average scores, the MPC profile had moderate scores, and the LPC profile had the lowest scores for each physical complaint indicator.

**Table 2:** Means and standard deviations of the five indicators of physical complaints associated with series watching across the three latent profiles identified by LPA: high physical complaints (HPC, *n* = 360, 24.1%), moderate physical complaints (MPC, *n* = 293, 19.6%), and low physical complaints (LPC, *n* = 843, 56.4%).

Variables	HPC		MPC		LPC		Test *(F)*	*p*
	*M*	*SD*	*M*	*SD*	*M*	*SD*		
PC1	3.08	0.91	2.02	1.06	1.29	0.61	**608.2**	^***^
PC2	3.11	1.03	2.01	1.14	1.35	0.71	**446.9**	^***^
PC3	2.88	1.20	1.85	1.10	1.34	0.72	**269.7**	^***^
PC4	2.94	1.24	2.08	1.16	1.41	0.82	**245.1**	^***^
PC5	3.08	1.26	2.21	1.28	1.49	0.83	**260.4**	^***^

M, mean; SD, standard deviation; Test = one-way analysis of variance; p = significance level. PC1: eye pain when watching series; PC2: blurred vision; PC3: watery eyes; PC4: dry eyes; PC5: headaches; ^***^p < 0.001. Bold values indicate statistically significant results (*p* < 0.05).

We analyzed the frequency pattern of watching three or more consecutive episodes of television series over the past 3 months (SW1) and found a significant association across the latent profiles (χ^2^ = 29.55; *p* < 0.001). The HPC profile had a lower proportion of participants reporting never engaging in this viewing pattern (12.8%) than the MPC (14.8%) and LPC (24.8%) profiles. Further, the HPC profile had more participants watching three or more consecutive episodes weekly or daily/almost daily, whereas the LPC profile showed a less frequent pattern of this behavior.

Notable differences in the number of episodes watched per session (question SW2) were observed across profiles (*F* = 9.15; *p* < 0.001). The HPC, MPC, and LPC groups watched averages of 3.37, 3.16, and 2.80 episodes, respectively. Additionally, the Games-Howell *post hoc* test showed that the HPC and MPC groups did not differ significantly, but both had higher mean scores than the LPC group.

The series-watching pattern was also significantly associated with auxiliary classification into the intensive series-watching (ISW) and less intensive series-watching (LSW) patterns, as indicated by a chi-square test (χ^2^ = 15.88, *p* < 0.001). The ISW group comprised the largest proportion of participants in the HPC profile (34.5%), followed by MPC (25.7%) and LPC (22.5%). Conversely, the LSW group was more prevalent in the LPC profile (77.5%) than in the MPC (74.3%) and HPC (65.5%) profiles, reflecting an association between higher physical complaints and a greater likelihood of being classified as an intensive series watcher.

Analyzing the sociodemographic characteristics of participants across the three profiles (Table 3) showed that all three profiles primarily included Brazilian participants; however, Canadian participants were proportionally more represented in the MPC and LPC profiles than in the HPC profile. Regarding gender, the HPC profile had more women, whereas the MPC and LPC profiles had more balanced gender distributions. Living arrangement and institution type were not significantly associated with the latent profiles. Regarding field of study, biological sciences represented the largest field in both the HPC and LPC profiles, whereas the MPC profile showed the highest proportion of students from social sciences.

**Table 3:** Sociodemographic characteristics across the three latent profiles of physical complaints associated with series watching identified by latent profile analysis (LPA): high physical complaints (HPC, *n* = 360, 24.1%), moderate physical complaints (MPC, *n* = 293, 19.6%), and low physical complaints (LPC, *n* = 843, 56.4%).

Variables	HPC		MPC		LPC		*X^2^*	*p*	Effect
	*N*	*%*	*N*	*%*	*N*	*%*			
Country							**13.29**	^***^	**0.10**
Brazil	310	86.1	228	77.8	652	77.3			
Canada	50	13.9	65	22.2	191	22.7			
**Gender**							**42.8**	^***^	**0.17**
Female	314	89.7	222	77.1	605	72.3			
Male	36	10.3	66	22.9	232	27.7			
**Who do you live with?**							**1.07**	**0.89**	**0.01**
Family members	245	71.4	202	73.5	586	72.8			
Roommates	48	14.0	40	14.5	108	13.4			
Alone	50	14.6	33	12.0	111	13.8			
**Institution type** ^***a***^							**2.46**	**0.29**	**0.04**
Private	303	97.7	224	98.2	629	96.5			
Public	7	2.3	4	1.8	23	3.5			
**Field of study** ^***a***^							**14.5**	^*^	**0.08**
Biological sciences	172	55.5	106	46.5	354	54.7			
Social sciences	128	41.3	115	50.4	251	38.8			
Applied sciences	10	3.2	7	3.1	42	6.5			

*N* = sample size; % = percentage; X^2^ = Chi-square test; *p* = significance level. The effect size was measured using Cramér's V. ^*^p < 0.05; ^***^p < 0.001.

^**a**^Institution type and field of study were collected only among Brazilian participants. Percentages were calculated from valid responses for each variable; therefore, totals may vary due to missing data. Bold values indicate statistically significant results (*p* < 0.05).

Table 4 displays the main emotional characteristics of the participants across the three profiles. For most of the variables analyzed, the participants in the HPC group scored significantly higher in food addiction, depression, anxiety, and stress, as well as lower quality of life. As SUPPS-P scores are inverted, lower SUPPS-P scores observed in the HPC profile indicate higher impulsivity-related indicators. Interestingly, the MPC profile generally showed intermediate scores between the HPC and LPC profiles, although this pattern was not observed across all variables. No statistically significant differences in profile were found for DERS Awareness, WHOQOL Social, SUPPS-P Lack of perseverance, and SUPPS-P Lack of premeditation.

**Table 4:** Main emotional characteristics across the three latent profiles of physical complaints associated with series watching identified by latent profile analysis (LPA): high physical complaints (HPC, *n* = 360, 24.1%), moderate physical complaints (MPC, *n* = 293, 19.6%), and low physical complaints (LPC, *n* = 843, 56.4%).

Variables	HPC		MPC		LPC		Test	*p*
	*M*	SD	*M*	SD	*M*	SD		
**mYFAS 2.0 (Total score)**	2.45	2.94	1.47	2.30	1.33	1.29	20.74	^***^
**AEBS (Total score)**	40.99	9.01	37.83	8.76	37.78	9.02	17.20	^***^
Appetitive drive	22.73	6.13	20.65	5.85	20.62	6.07	16.25	^***^
Low dietary control	18.25	4.56	17.18	4.49	17.16	4.62	7.75	^***^
**SAS-SV (Total score)**	37.80	10.19	33.22	10.01	30.94	10.08	57.62	^***^
**DERS (Total score)**	50.73	13.75	45.15	14.58	42.13	13.59	49.8	^***^
Awareness	7.39	2.96	6.88	2.67	7.24	2.97	2.85	**0.06**
Clarity	8.26	3.27	7.37	3.00	7.01	3.11	18.88	^***^
Goals	11.09	3.20	9.97	3.75	9.00	3.63	50.31	^***^
Impulse	7.49	3.77	6.42	3.46	5.67	3.10	33.74	^***^
Nonacceptance	8.09	3.85	7.47	3.78	6.70	3.55	18.53	^***^
Strategies	8.41	3.82	7.04	3.55	6.51	3.46	32.89	^***^
**DASS-21 (Total score)**	33.35	14.30	26.89	14.56	22.61	14.09	72.33	^***^
Depression	10.66	5.67	8.52	5.72	7.32	5.36	45.49	^***^
Anxiety	10.60	5.43	8.21	5.45	6.59	5.24	70.90	^***^
Stress	12.08	4.79	10.16	4.86	8.70	4.92	62.69	^***^
**WHOQOL (Total score)**	87.04	14.54	90.76	14.16	92.88	14.15	19.12	^***^
Physical	23.47	4.59	24.69	4.69	25.57	4.20	26.04	^***^
Psychological	18.35	4.22	19.53	4.37	20.07	4.36	18.92	^***^
Social	10.42	2.46	10.46	2.61	10.67	2.53	1.44	**0.23**
Environmental	28.30	5.35	29.21	5.05	29.45	5.24	5.39	^*^
**SUPPS-P (Total score)**	54.21	7.71	55.25	6.68	56.05	7.45	6.92	^**^
Negative urgency	9.76	2.92	10.64	2.79	10.78	2.96	14.55	^***^
Lack of perseverance	11.70	1.66	11.77	1.61	11.61	1.54	1.17	**0.31**
Lack of premeditation	11.12	1.75	11.27	1.70	11.25	1.64	0.74	**0.48**
Sensation seeking	10.16	2.84	9.63	2.87	10.23	2.89	4.64	^*^
Positive urgency	11.47	3.13	11.93	2.78	12.18	3.00	6.13	^**^

M, mean, SD, standard deviation, Test = one-way analysis of variance, *p* = significance level. ^*^p < 0.05; ^**^p < 0.01; ^***^p < 0.001. Bold values indicate statistically significant results (*p* < 0.05).

**Figure 1:**
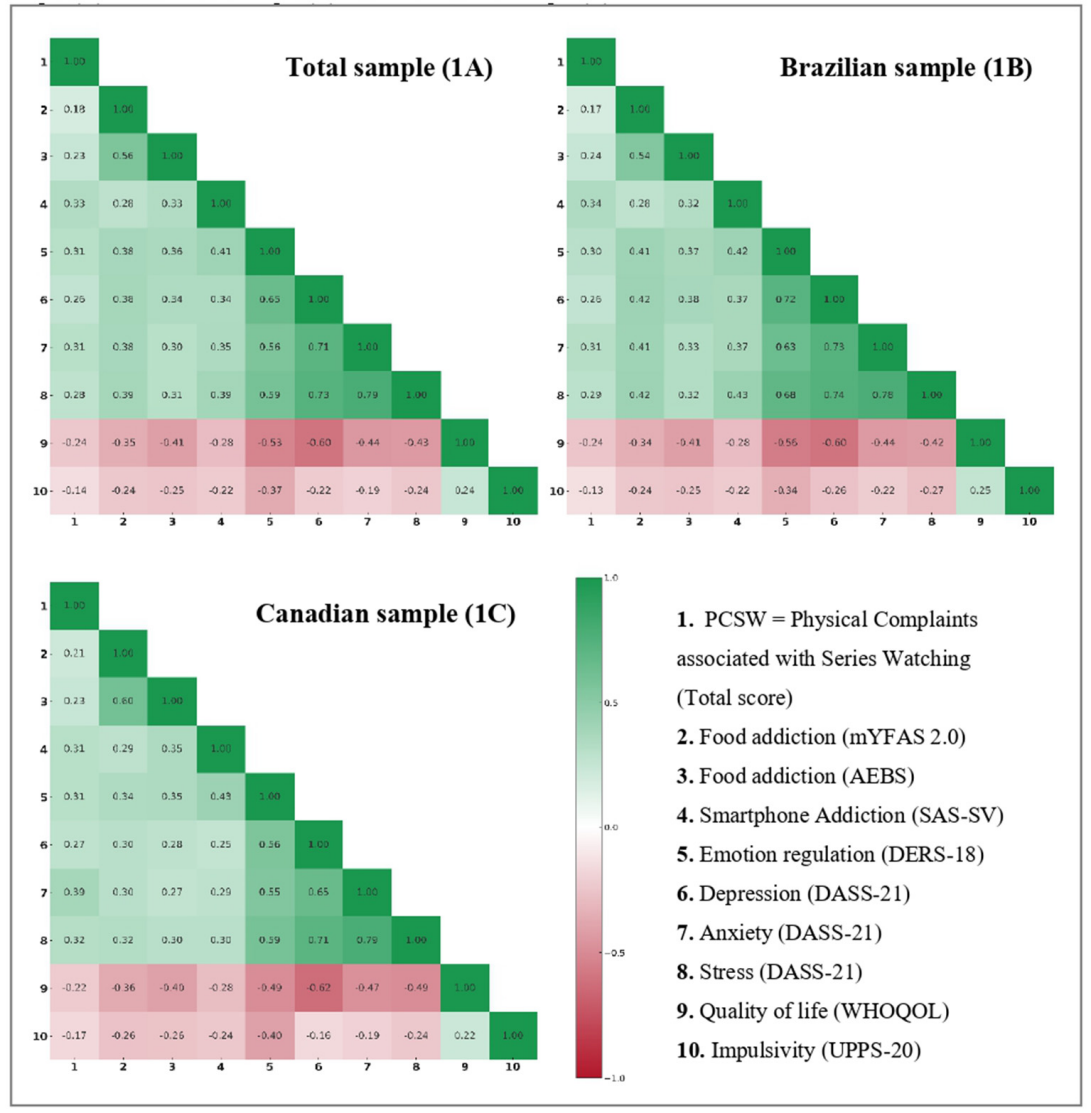
Heatmap showing Spearman's correlation coefficients among study variables for the overall sample **(A)**, the Brazilian sample **(B)**, and the Canadian sample **(C)**. *Note*. The heatmaps display the Spearman rank-order correlation coefficients between study variables. Green cells indicate positive correlations, while red cells represent negative correlations. Darker shades indicate stronger associations. Panel A presents the correlations for the general sample, Panel B for the Brazilian sample, and Panel C for the Canadian sample.

Regarding correlations (Figure 1), in all analyses (full sample 1A, Brazilian sample 1B, and Canadian sample 1C), distress/psychopathology (DASS-21) and emotion regulation difficulties (DERS-18) formed a highly interconnected cluster. PCSW, smartphone addiction, and food addiction were positively linked to this cluster. The SUPPS-P instrument showed negative correlations with PCSW and other risk factors, consistent with theoretical expectations. Quality of life was negatively related to nearly all distress variables and to PCSW. The country-specific matrices exhibited generally similar patterns, though not identical. While some variability in coefficient magnitude was noted between the Brazilian and Canadian samples, these differences were interpreted descriptively.

**Figure 2:**
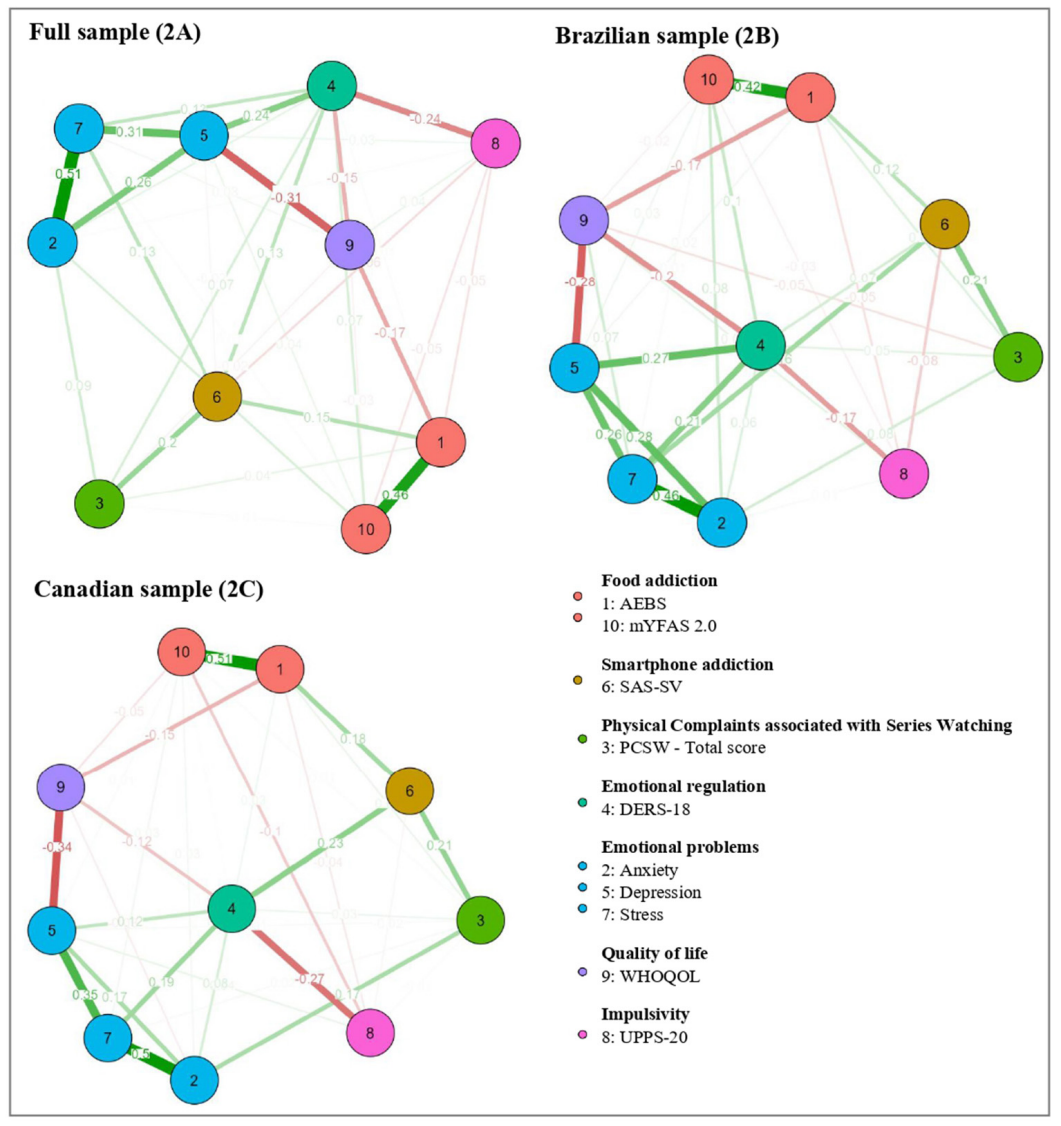
An exploratory Gaussian graphical model derived from network analysis, including multiple variable types. Panel A displays the full sample; Panel B shows only Brazilian participants; and Panel C shows only Canadian participants.

Figure 2 shows three networks from the exploratory Network Analysis (NA): the total sample (2A), Brazilian students (2B), and Canadian students (2C). The networks suggested a broadly similar descriptive organization in which anxiety, depression, and stress formed a core cluster connected to emotion regulation difficulties, while quality of life showed negative associations with these nodes. PCSW was positively related to smartphone and food addiction indicators and negatively related to quality of life. Impulsivity showed negative connections with PCSW and emotional variables. Country-specific networks indicated differences in the strength of certain associations but did not include formal tests for network invariance. In Brazil (Figure 2B), the connections between PCSW and smartphone addiction, as well as between PCSW and emotion regulation, seemed stronger based on description. In Canada (Figure 2C), although the general pattern resembled that of Brazil, food addiction indicators were more salient with respect to PCSW and emotional variables. These findings should be viewed with caution due to unequal sample sizes across countries.

Centrality measures from the Network Analysis (Table 5) were interpreted descriptively. In the full sample, depression, stress, and difficulties with emotion regulation showed the highest centrality indicators and comprised the main core of the network. Depression showed the highest closeness and strength, stress showed high strength and the strongest expected influence, and emotion regulation showed elevated coefficients for both closeness and strength. Quality of life occupied a central negative position, with a strongly negative expected influence coefficient. PCSW appeared to be connected to the network, but with an expected influence value close to zero. For Brazilian students, stress was among the most relevant nodes, showing the highest betweenness, strength, and expected influence, followed by emotion regulation and depression. We also observed that problematic smartphone use had relatively high betweenness, suggesting a possible connecting role between PCSW and emotional variables. Because no formal bootstrap stability analysis was performed, centrality-based interpretations should be treated as exploratory.

**Table 5:** Descriptive centrality coefficients for the entire sample and by country (Brazil vs. Canada).

Variables	Full sample			
	Betweenness	Closeness	Strength	Expected influence
AEBS	0.808	−0.261	0.196	0.091
Anxiety	−0.808	0.356	0.576	1.127
PCSW	−1.078	−1.548	−1.521	−0.003
DERS-18	1.078	0.745	0.954	−0.173
Depression	1.347	1.451	1.399	0.294
SAS-SV	0.539	0.388	−0.558	0.256
Stress	−0.539	0.630	1.037	1.354
SUPPS-P	−1.078	−1.306	−1.335	−1.372
WHOQOL	0.808	0.607	−0.352	−1.923
mYFAS 2.0	−1.078	−1.061	−0.396	0.349
Brazilian sample	
AEBS	−0.027	−0.445	0.044	−0.009
Anxiety	−0.846	0.503	0.486	1.069
PCSW	−1.119	−1.672	−1.369	−0.133
DERS-18	0.518	0.895	1.057	−0.030
Depression	0.518	1.264	1.131	0.338
SAS-SV	1.064	0.108	−0.640	0.139
Stress	1.610	0.986	1.245	1.482
SUPPS-P	−1.119	−1.238	−1.521	−1.304
WHOQOL	0.518	0.415	0.033	−1.917
mYFAS 2.0	−1.119	−0.817	−0.466	0.366
Canadian sample	
AEBS	0.992	−0.440	0.537	0.373
Anxiety	−0.558	−0.120	0.641	0.995
PCSW	−1.178	−0.853	−1.376	0.204
DERS-18	0.992	1.539	1.109	−0.133
Depression	0.682	0.548	0.968	0.009
SAS-SV	0.372	0.505	−0.773	0.441
Stress	0.992	1.322	1.145	1.343
SUPPS-P	−1.488	−1.053	−1.394	−1.422
WHOQOL	0.372	0.062	−0.528	−1.943
mYFAS 2.0	−1.178	−1.510	−0.330	0.133

## 4 Discussion

We aimed to identify latent profiles of PCSW and examine differences across profiles in emotional variables, problematic smartphone use, dysfunctional eating patterns, quality of life, and SWI among university students from Brazil and Canada. Overall, the findings largely supported the proposed hypotheses. LPA identified three profiles characterized by high, moderate, and low levels of physical complaints: HPC, MPC, and LPC. The HPC profile showed the most adverse pattern, with higher levels of emotional symptoms, emotion-regulation difficulties, problematic smartphone use, addictive-like eating indicators, lower quality of life, and greater SWI. This pattern aligns with recent latent profile research indicating that problematic digital behavior profiles can vary significantly in distress indicators, fear of missing out, and self-esteem (Aslan and Koç, 2026). Spearman correlations also demonstrated positive associations between PCSW and emotional distress, emotional dysregulation, problematic smartphone use, and addictive-like eating indicators, whereas quality of life was negatively associated with PCSW and the other risk indicators. Finally, the exploratory network analysis suggested a descriptive organization in which stress, depression, and emotion-regulation difficulties occupied central positions, while PCSW was more peripherally connected to this structure, especially through problematic smartphone use and emotional dysregulation. Because no formal bootstrap stability analysis was performed, these network findings should be interpreted cautiously and treated as exploratory.

The differences among the three latent profiles were pronounced, particularly regarding the PCSW burden. The HPC profile showed the highest scores across the five indicators of physical complaints, including eye pain, blurred vision, tearing, dry eyes, and headaches, followed by the MPC and LPC profiles. In practice, higher PCSW was associated with poorer visual wellbeing and lower quality of life, consistent with prior evidence linking prolonged screen exposure to ocular discomfort and digital eye strain. Extended screen time, possibly more prevalent among students with higher PCSW, may be associated with digital eye strain, including eye strain, asthenopia, blurred vision, tearing, dry eyes, and headaches (Allwihan et al., 2024; Kaur et al., 2022; Pucker et al., 2024). However, because the present study was cross-sectional, these findings should not be interpreted as evidence that binge-watching caused the physical complaints.

We also observed an association between PCSW and SWI, as the HPC profile showed a higher intensity of series watching than the LPC. Similarly, a recent systematic review associated prolonged screen use, especially ≥ 8 h/day, with symptoms of digital eyestrain and dry eye disease (Kopilaš et al., 2025). These authors also observed that such symptoms were associated with depression, anxiety, and stress. Initially, PCSW may appear limited to direct effects, such as eye problems and headaches related to extended screen time. However, our findings suggest that PCSW is also connected to other areas of life, as indicated by mental health and addictive-like eating indicators. Previous studies have shown that SWI may be associated with unhealthy eating patterns, particularly among young people (Aghababian et al., 2021; Dejavitte et al., 2025; Garg et al., 2025; Starosta et al., 2020). This study's HPC profile, which showed the highest PCSW and greater SWI, also had higher mYFAS 2.0 scores than the other profiles, suggesting an association between physical complaints related to series watching and addictive-like eating indicators. Additionally, the AEBS results indicated more frequent addiction-like eating patterns in the HPC profile. This finding aligns with recent research involving Turkish university students, which indicates that problematic digital media use is linked to food addiction, eating behaviors, and indicators related to obesity (Toguç, 2026). These findings should be understood dimensionally, not as evidence of clinical food addiction.

Problematic smartphone use also emerged as another behavioral correlate of PCSW, as reflected by a higher HPC profile score on the SAS-SV. In the heatmap, smartphone addiction showed one of the strongest links with PCSW. In the exploratory network analysis, this same variable appeared to occupy an intermediate position between PCSW and the emotional distress core, including anxiety, depression, and stress. This pattern suggests that problematic smartphone use may be important for understanding how PCSW relates to emotional symptoms, although no mediational or causal pathway was specifically tested. These findings suggest that notifications and other smartphone-based features may indirectly facilitate more frequent series watching and related emotional burden (Kaur and Ashfaq, 2023; Raza et al., 2021; Sever and Özdemir, 2021). The ability to subscribe to and access streaming services through mobile apps has enabled portable series-watching across various settings (Chen et al., 2024; Starosta et al., 2020). Additionally, companies have notably adapted to smartphone features, including sending notifications and recommending new content based on the user's previously watched genres and stories (Romero Meza and D'Urso, 2024). Thus, problematic smartphone use should be understood as a dimensional indicator of risk-related digital behavior rather than as a formal clinical diagnosis.

Regarding quality of life, the analysis of profiles derived from LPA showed that PCSW was associated with lower quality-of-life scores; the HPC profile had the lowest overall WHOQOL scores and the lowest scores in the physical and psychological domains. Heatmaps showed that quality of life was negatively linked to PCSW and emotional factors; additionally, the exploratory network analysis indicated that quality of life had a strong negative expected influence. This finding reinforces the inverse association between quality of life and indicators of emotional or behavioral vulnerability in the present sample. Previous studies have also shown that SWI may be associated with reduced wellbeing, increased stress and anxiety, and difficulties with emotion regulation (Castro et al., 2021; Pittman and Steiner, 2021). Additionally, the connection between emotion-regulation difficulties and impulsivity is relevant in this context, as both have been associated with SWI behaviors and other addictive-like behaviors (Diotaiuti et al., 2022; Flayelle et al., 2020; Srinivasan et al., 2021; Starosta et al., 2020). In this study, this pattern should be interpreted with the SUPPS-P's reversed scoring, in which lower scores indicate higher impulsivity-related indicators. It is also important to note that not all indicators of quality of life and impulsivity showed significant differences between profiles, underscoring the need for a nuanced interpretation of these results.

The HPC profile showed higher anxiety, stress, and depression levels. In Spearman's correlations, PCSW was positively correlated with emotional symptoms and difficulties in emotion regulation, and negatively correlated with quality of life. In the exploratory network analysis, these variables formed the main descriptive core of the structure, whereas PCSW was mainly connected to them through problematic smartphone use and emotional dysregulation. Some authors note that low mood can lead to greater SWI, as people seek ways to avoid negative symptoms; however, increased screen time can worsen behaviors typically associated with depression, such as neglecting daily tasks and social isolation (Bastos et al., 2024; Steins-Loeber et al., 2020). A similar process may occur with anxiety and stress, as symptom relief, relaxation, or emotional avoidance may motivate more intense series watching (Alimoradi et al., 2022). However, it is important to note that prolonged and isolating patterns of series watching may also be associated with social interaction anxiety and loneliness, due to extended periods of reduced face-to-face interaction and increased screen time (Sun and Chang, 2021). Regarding stress, connections can be made with digital stress, which results from excessive use of Information and Communication Technologies and causes anxiety, insomnia, sleep disorders, aggressiveness, and psychosomatic symptoms, significantly reducing quality of life (Lin and Su, 2025). Given the cross-sectional design, these relationships should be interpreted as associations rather than directional pathways.

From a cross-cultural perspective, the growth of streaming platforms in Brazil is closely linked to the expansion of smartphone use and digital media consumption (da Silva et al., 2025). Although countries such as Canada have greater access to digital technologies, digital media use or its emotional and behavioral correlates may not be uniform across contexts. This pattern was reflected in the profiles identified in this study: all profiles were predominantly composed of Brazilian participants, but Canadian participants were proportionally more represented in the MPC and LPC profiles than in the HPC profile. In addition, the exploratory country-specific networks suggested broadly similar, although not identical, descriptive patterns across countries. In Brazil, associations between PCSW, problematic smartphone use, and emotion regulation seemed more pronounced, while in Canada, indicators of addictive-like eating were more prominently linked to PCSW and emotional factors. These findings suggest that PCSW and its emotional and behavioral correlates may follow a similar general organization across countries, while still showing context-specific differences in the magnitude and role of some variables. Since no formal measurement invariance or network comparison tests were performed, and given the unequal sample sizes of the Brazilian and Canadian samples, cross-country differences should be interpreted with caution, primarily as descriptive.

In the university context, other relevant variables include the type of institution and the area of study. Some studies have discussed how series-watching patterns may vary across academic contexts and students' routines (Ramayan et al., 2018). In this study, the HPC profile included a large proportion of students from private institutions; however, institution type was not significantly associated with the latent profiles, and this finding should be interpreted as a characteristic of the present sample rather than evidence of a consistent institutional effect. The field of study showed a significant, although small, association with the profiles, with a stronger presence of students from biological sciences in the HPC and LPC profiles, whereas the MPC profile had a higher proportion of students from social sciences. This may be relevant because students in health-related areas are often exposed to high academic demands and stress, which may interact with screen use, emotional symptoms, and physical complaints (Hamza et al., 2023). However, this interpretation should be approached with caution, as the present study was not designed to determine whether specific academic fields increase the risk of PCSW.

Although this study contributes to understanding PCSW and its emotional and behavioral correlates among university students, it has some limitations. First, because of its cross-sectional design, causal relationships cannot be established. In addition, PCSW was assessed using self-report items, as no validated scales exist to measure physical complaints associated with series watching in Brazil or Canada. Therefore, the PCSW construct should be understood as an operational measure based on theoretically related items. While this operational approach was effective in identifying exploratory latent profiles, future research should further investigate the psychometric properties of these items, including their dimensionality, reliability, and measurement invariance across countries. The use of convenience sampling limits the generalizability of the findings, and the unequal sample sizes between Brazil and Canada should be considered, particularly when interpreting country-specific network patterns. Moreover, the network analysis was exploratory and did not include formal bootstrap stability estimates, edge-weight confidence intervals, centrality-stability coefficients, or formal tests of network invariance. For this reason, centrality-based and country-specific interpretations should be regarded as descriptive. Finally, all measures were self-reported, and the study did not include objective indicators of screen time, device use, ocular health status, or clinical assessment of visual complaints.

We suggest that future studies use longitudinal designs to examine how PCSW, SWI, emotional symptoms, smartphone use, and quality of life interact over time. The mediating and moderating roles of impulsivity, coping strategies, self-esteem, and social support could also be tested within the I-PACE model of behavioral addictions (Brand et al., 2025). In addition, objective measures of screen time and usage patterns, such as app-based monitoring, may complement self-report data and improve measurement accuracy. Future research should focus on developing and validating specific tools to assess physical complaints related to series watching and examine whether the structure of PCSW is consistent across different cultural contexts.

## Data Availability

The raw data supporting the conclusions of this article will be made available by the authors, without undue reservation.
